# Pathological and Clinical Observations on the Fœtid Grains or Tubercles, Mixed with the Sputa of Consumptive Persons

**Published:** 1806-01

**Authors:** 


*Pathological and Clinical Observations on the fœtid Grains or Tubercles, mixed with the Sputa of consumptive Persons. By* Dr. Ballhorn.

It is frequently observed, that persons, apparently in good health, expectorate little whitish grains or globuli, like a small pea, and even less, similar to spermaceti, which when they are rubbed in pieces cause a disagreeable smell. Sometimes this matter is more fluid, sometimes more glutinous, and it is excreted during spitting, or coughing, or calling aloud. In general, it is considered a symptom preceding consumption; it is therefore the more to be wondered that no inquiries have been made to investigate the cause of this phenomenon. When the expectoration of these grains is accompanied with cough, dyspnœa, and pain in the chest, the origin may be justly deemed to proceed from the lungs, and it often happens that the spitting of blood supervenes, and the patient dies of a consumption; but, on the contrary, there are many instances of people being affected with this accident, enjoying in every respect a perfect good health: we are then left to conclude, that the seat of this evil cannot always be the same; and it seems very probable that the coagulum sometimes is formed in the lungs and bronchiæ, when it is expectorated in coughing; in the throat, when it is eliminated by calling aloud; or in the larynx. On minute examination, we find *that this coagulum has the greatest similarity with the tartar adhering to the teeth*; and it may be observed, that persons, whose teeth are affected in such manner, are likewise liable to such an expectoration. Dr. B. therefore draws the conclusion, that in both ways the origin is the same, viz. the bad state of the stomach and the organs of digestion.

But whether this tartar of the teeth, the fauces, or the pneumatic organs, may be considered as a crude matter of diseased digesting organs, deposited in these places, and formed from corrupted juices; whether it is a more animalized and secreted matter from the blood; or whether it may be thought to be a precipitate from the spittle deposited on the gums, or from the exhalations of the bronchial glands deposited on the fauces or the bronchiæ, it cannot be evinced with certainty; but, beyond doubt, an anomalous chylification contributes mostly towards its formation.

It

It is of the highest importance when any patient expectorates such a substance, to examine the seat of it. When there is no difficulty of breathing, no stitches in the chest, no cough, nor difficult expectoration, the matter probably is secreted in the fauces, and this accident is more tiresome than dangerous. But if we observe the formation to take place in the lungs, we cannot draw the absolute conclusion that consumption will follow. How many persons die of old age or other accidents, whose lungs were deemed perfectly sound till, after death, dissection has proved the contrary? In the following instances only, this coagulum seems to produce a consumptive state.

1. When a real calcareous or earthy matter is deposited in the bronchiæ; as long as this matter remains soft and friable, it may be removed by coughing or expectorating. In this case the asthma is not constant, but more periodically; sometimes it entirely vanishes; but when the soft matter is rendered more calcareous, and the pneumatic tubes are obstructed, then the asthma is constant, and will not yield to the most efficacious remedies; if the lungs are not very irritable, it may be borne perhaps a long time, even without injury.

2. When the circulation in the lungs has been impeded by these obstructions, the tartar causes a violent irritation, cough, and hæmoptysis; and a chronic inflammation is brought on, ending in consumption and hectic.

That in such tartarised lungs, spitting of blood may easily be produced, is proved by analogy. Persons whose teeth are covered with tartar, are liable to bleeding from the gums. Is it not probable then that the tartar, collected in the lungs or bronchiæ, may debilitate and irritate the blood vessels of the lungs, so as to produce hæmoptysis? However, the debilitating power of the tartar cannot have the same effect as upon the gums, being much less exposed to the air.

Now, it may be asked, whether there are any remedies which possess the power of dissolving the tartarized concretions? and whether this species of consumption may admit of a cure? This, in general, cannot be answered. It depends on the structure and formation of these concrements. Dr. B. mentions several instances where the expectoration being mixed for a great length of time with such grains and globuli as have been described above, hæmoptysis soon after ensued, and all the other symptoms of of consumption; nevertheless, the patients perfectly recovered, and enjoyed a durable state of health. But when the soft tartar is reduced to an earthy or calcareous matter, it cannot be absorbed or thrown out by coughing. And yet, considering the great power of the absorbents, even to take up and to emolliate bones, the absorbing power of the lungs cannot be entirely denied; and our endeavours would be in some measure rewarded, if at least some portion of the concrements was to be removed by absorption, or even if a new formation or reproduction could be prevented. It is beyond doubt, and has been proved by pathological dissections, that such patients may attain a great age.

The method of cure therefore must be chiefly directed to prevent the production and increase of the concrements; in general, the progress will be slow, but with a careful and assiduous perseverance will now and then succeed. Sometimes the way of living, and the diet of the patient, suddenly put a stop to the progress; for instance, a milk diet is exceedingly hurtful.

The Doctor lays down the following plan, and list of medicines, by the continuance of which the patient will find himself considerably relieved. The diet must be light and nourishing; all the acids, wine, spirituous liquors, milk, cheese, and fat, should be carefully avoided.

Lime water has, in many instances, been the most serviceable.

3. Thc digitalis purpurea, as much as the patient can bear, on account of the connection between the lungs and kidnies.

4. Ipecacuanha in small doses, and often repeated, but not so far as to create nausea. The Doctor thinks it relieves in small doses the spastic affections, which are often associated with this complaint.

5. Sulphur stibiatum aurantiacum, in one or two grains, is a useful medicine to promote gentle expectoration.

6. The light mineral waters of Seltz, Embs, &c. which contain alkaline particles.

7. Bark, quassia, rhubarb in small portions, and other bitters; and assafœtida for promoting and strengthening the organs of digestion and chylification.

8. All kind of passive exercise, as riding on horseback, swinging, &c.

